# Rhamnolipid Biosurfactant: Use for the Removal of Phenol from Aqueous Solutions by Micellar Solubilization

**DOI:** 10.1021/acsomega.3c04367

**Published:** 2023-08-09

**Authors:** Victor P. Arkhipov, Ruslan Arkhipov, Andrei Filippov

**Affiliations:** † Department of Physics, Kazan National Research Technological University, Kazan 420015, Russian Federation; ‡ Institute of Physics, Kazan Federal University, Kazan 420008, Russian Federation; § Chemistry of Interfaces, Luleå University of Technology, 971 87 Luleå, Sweden

## Abstract

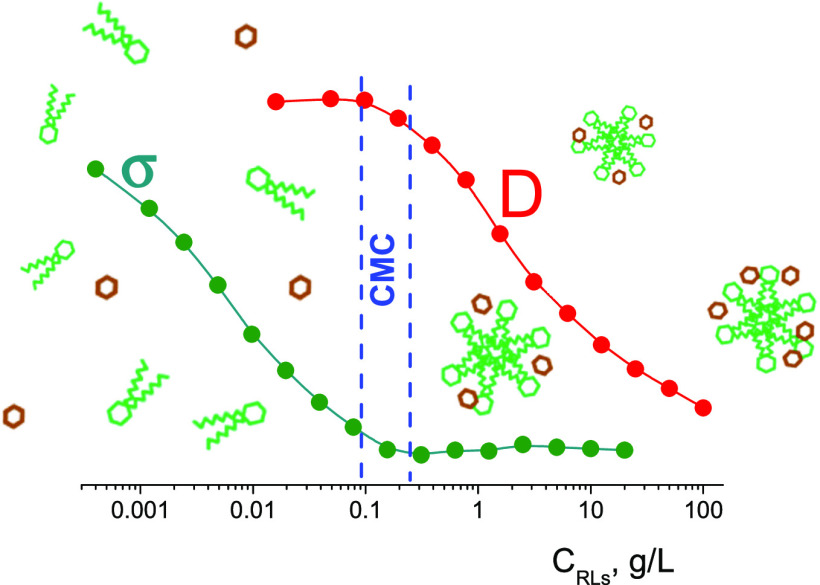

Selective measurements of the self-diffusion coefficients of molecules of the biological surfactant rhamnolipid (RL) in individual aqueous solutions and in solutions with phenol as a solubilizate were carried out by nuclear magnetic resonance (NMR) diffusometry. Based on the obtained results, the solubilization characteristics of RLs were calculated. They are the fraction of solubilized phenol molecules, the phenol micelle–water distribution coefficient, the molar solubilization coefficient, the hydrodynamic radii of RL monomers and micelles, the aggregation numbers of micelles, and the solubilization capacity of micelles. Fraction of the solubilized phenol molecules increases and approaches 80–90% with increasing RL concentration. The solubilization capacity of the micelles increases from several units to 10^2^ with an increase in both the concentration of RLs and the concentration of phenol in solution.

## 1 Introduction

Due to their low toxicity, high degree of biodegradability, and low cost, biological surfactants^1−3^ produced by living microorganisms are considered an attractive alternative to synthetic surfactants, whose widespread use adversely affects the environment. The most extensively studied types of biosurfactants are rhamnolipids (RLs) and sophorolipids.

**Figure 1:**
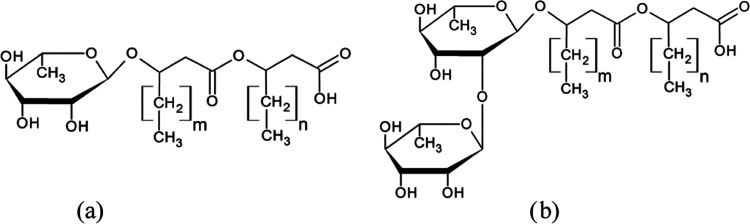
Molecular structure of (a) mono-rhamnolipid and (b) di-rhamnolipid.

Rhamnose lipids or rhamnolipids are biological anionic surfactants^4,5^ belonging to a class of glycolipids produced by *Pseudomonas aeruginosa* bacteria. Rhamnolipids contain a hydrophilic head of one or two rhamnose groups and a hydrophobic tail of one or two 3-hydroxy fatty acids chains (Figure 1).

In aqueous solutions, at concentrations *C* higher than the critical micelle concentration (CMC), rhamnolipids form micelles^6^ capable to solubilize organic and inorganic impurities: hydrocarbons,^7^ heavy metals,^8^ Cd,^9^ and Cr.^10^ Rhamnolipids are used in soil remediation technologies^11,12^ after oil and oil product spills and wastewater treatment.^9,13^

In our previous work,^14^ micellar solubilization by rhamnolipids of aromatic hydrocarbons, poorly soluble in water (benzene, toluene, ethylbenzene, and xylene (a BTEX group)), was studied. In this work, we use the capabilities of NMR diffusometry to study micellar and solubilizing properties of rhamnolipids in aqueous phenol solutions. Unlike substances of the BTEK group, whose solubility in water is about 0.1 g/L, the solubility of phenol is much higher, 65 g/L at 298 K. It has been shown that at high RL concentrations, the fraction of solubilized BTEX molecules is close to 100%, i.e., almost all BTEX molecules are in the solubilized state in RL micelles. The aromatic ring and hydroxyl group cause phenol to have chemical properties that are characteristic of both alcohols and aromatic hydrocarbons. Will phenol be solubilized by rhamnolipid micelles and to what extent?

Phenols are dangerous and, unfortunately, widespread anthropogenic environmental pollutants.^15^ Of particular danger are the waste waters of oil refineries, coke-chemical enterprises, pulp and paper mills, production of building materials, rubber, adhesives, plastics, pesticides, phenol–formaldehyde resins. The maximum permissible concentration (MPC) of phenols in reservoirs and drinking water is strictly regulated,^16^ with the content of phenol not to exceed 0.001 mg/dm^3^. Phenols, like other organic compounds of the aromatic series, are detrimental to many microorganisms, so industrial wastewater is difficult to treat biologically.

For the treatment of industrial wastewater, enzymatic destruction of phenol by bacteria, catalytic oxidation and ozonation, extraction, membrane, filtration, and adsorption methods are used.^17^ Extraction methods are based on surfactants as extractants can be divided into methods based on cloud point extraction^18^ and methods using the micellar solubilization phenomenon.^19^ The micellar extraction method is based on the property of surfactant micelles to solubilize organic compounds, metals, and other pollutants.

In this work, we studied the solubilization properties of RL micellar solutions with respect to the simplest representative of the phenol class, hydroxybenzene C_6_H_5_OH. The efficiency of the solubilization process and the solubilization characteristics of RLs (micelle–water partition coefficient *K*_m_, molar solubilization ratio MSR) were calculated based on the results of selective measurements of the self-diffusion coefficients of phenol, RLs, and water molecules, performed by NMR diffusometry methods.^20^ The self-diffusion coefficients of molecules of individual components, micelles, and aggregates make it possible, based on the Stokes–Einstein relation,^21^ to draw conclusions about the size, shape, and composition of diffusing objects. In particular, the solubilization of phenol molecules or any other extractable compound by surfactant micelles unambiguously manifests itself in the fact that their diffusion coefficients *D*s begin to approach and coincide with each other at complete solubilization. Experimental problems may arise if the NMR spectral lines of the extracted compound and the surfactant overlap, in which case measurements of diffusivities on various magnetic nuclei or special mathematical methods for processing NMR spectra can help.^22^

All experimental procedures, including recording of NMR spectra, measurement of relaxation time *T*_1_, measurement of diffusion coefficients of components, and CMC measurements, were performed at a constant temperature of 25 °C (298 K). The measurements were carried out at four concentrations of phenol *C* = 10, 5, 1, and 0.1 g/L depending on the concentration of RLs from 200 to 0.05 g/L in an aqueous solution. Tensiometry and diffusometry methods were used to determine the CMC of aqueous solutions of RLs.

## 2 Results and Discussion

### 2.1 Critical Micelle Concentration of RLs

**Figure 2:**
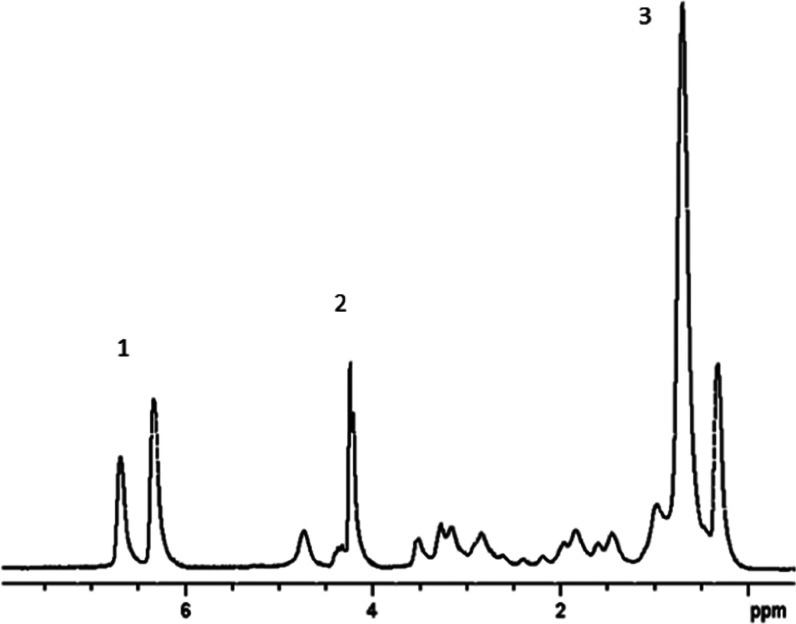
^1^H NMR spectrum of the RLs in aqueous D_2_O solution in the presence of phenol: (1) lines of aromatic protons of phenol (δ = 6.7, 6.3 ppm), (2) the line of residual protons of water (δ = 4.2 ppm), and (3) the line of methylene protons of rhamnolipid (δ = 1.2 ppm). *T* = 298 K.

In the spectrum of the system under study, Figure 2 contains lines of aromatic protons of phenol (δ = 6.7; 6.3 ppm), residual protons of water (δ = 4.2 ppm), and lines of RLs. The self-diffusion coefficients of rhamnolipid molecules were determined from the decays of the integral intensities of the methylene group proton lines (δ = 1.2 ppm).

Results of measuring the CMC of rhamnolipids in aqueous solutions by tensiometry, viscometry, and conductometry, which are based on sharp changes in the surface or bulk properties of surfactants during the transition from the molecular to micellar state of surfactants in solution, are inconsistent with each other. The CMC values of mono- and di-rhamnolipids and their mixtures, obtained by various methods, range from 1 to 400 mg/L.^23−25^ In our previous work,^14^ we obtained the value of CMC = 0.35 g/L using diffusometry and conductometry methods. Bearing in mind the ambiguity and significant scatter of the above values, we performed separate independent measurements of CMC in order to more accurately characterize the samples under study.

**Figure 3:**
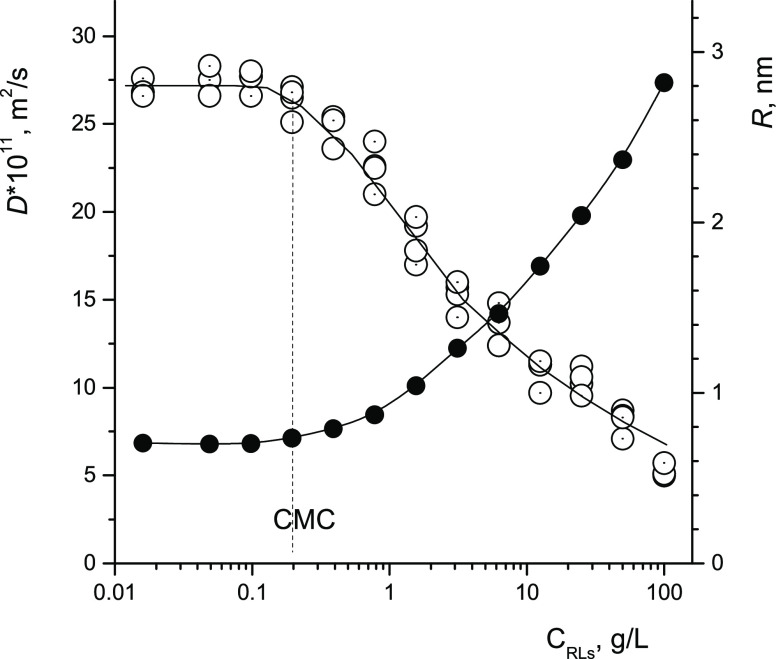
Coefficients of self-diffusion of molecules and radii of monomer and micelle RLs depending on its concentration in an aqueous D_2_O solution at 298 K (the results of several independent series of measurements are presented). Diffusion coefficients are open symbols; radii of monomers at *C* < CMC and micelles at *C* > CMC are closed symbols.

A decrease in the mobility of surfactant molecules during the transition from the monomeric to the micellar state and, accordingly, a sharp decrease in the diffusivity of surfactant molecules are used in NMR methods for determining CMC.^20,26−28^ The results of measuring diffusion coefficients of RL molecules in individual aqueous solutions are shown in Figure 3. Diffusion coefficients were determined from the initial segments of diffusion decays. The inflection point of the dependence of the effective *D* on the concentration of rhamnolipid in solution, corresponding to *C* = CMC, is observed at *C* ≈ 0.20 g/L.

For a clearer idea of the changes occurring in RL solutions, we calculated the effective hydrodynamic radii *R* of monomer and micelle RLs with corrections^29^ for their interaction with each other using the Stokes–Einstein relation^21^ and the measured values of *D*s of RLs
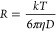
1where *k* is the Boltzmann constant and η is the dynamic viscosity of the solvent. The value of the coefficient of dynamic viscosity of heavy D_2_O water at 298 K is taken as equal to 1.138 mPa·s.^30^ The results presented in Figure 3 correspond to some average particle radii because the sample under study is a mixture of mono-RL and di-RL. At *C* < CMC, only RL monomers with average effective hydrodynamic radii ≈0.7 nm are present in the solution. At *C* > CMC, micelles are formed, the sizes of which grow with an increase in the concentration of RLs in the solution, reaching 3 nm at *C* = 100 g/L. An increase in the size of RL micelles at *C* > CMC and a change in their shape from spherical to sphero-cylindrical and worm-like has been reported based on DLS measurements,^31^ SANS,^32^ surface tension, dynamic light scattering, and both steady-state and time-resolved fluorescence spectroscopy.^33^ In mixed micelles, the change in the shape of micelles is noted depending on the ratio of mono- and di-RLs.^34^

**Figure 4:**
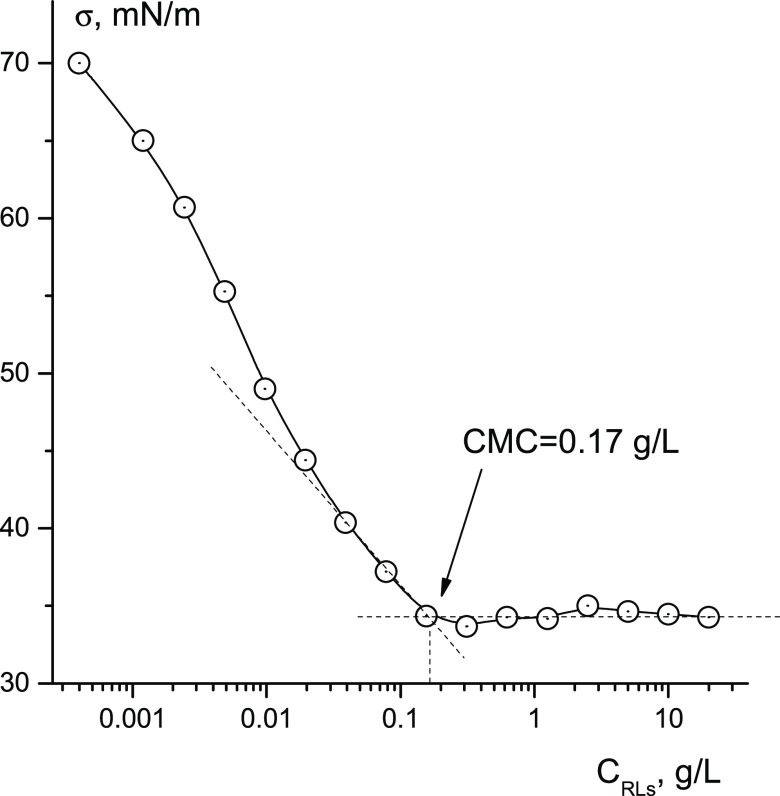
Coefficient of surface tension of aqueous H_2_O solution RLs depending on the concentration of RLs in the solution. *T* = 298 K.

The determination of the CMC by changing the surface properties of surfactant solutions is based on the termination of the change in the surface tension^35^ of the solution at the limiting saturation of the adsorption layer. The results of measuring the surface tension of RL solutions depending on the concentration of RLs in the solution are shown in Figure 4. Measurements made on a digital tensiometer using the ring tear-off method, with five repetitions at each concentration, give a CMC value of ≈0.17 g/L. It can be noted that the CMC values obtained by independent methods of diffusometry and tensiometry are in good agreement with each other.

### 2.2 Solubilization of Phenol by Micelles of RLs

RL micelles solubilize phenol, and the efficiency of solubilization can be calculated from changes in the *D*s of phenol molecules depending on the concentration of RLs in solution. Specifically, if there is no solubilization, then the phenol *D* values should not depend on the presence of RLs in the solution; a possible decrease in the phenol *D*s can only be associated with a change in the dynamic viscosity of the solution with an increase in the RL concentration. When phenol molecules are solubilized by RL micelles at *C* > CMC or by RL premicelles at *C* ≈ CMC, one should expect a decrease in the *D* values of phenol, their convergence with the values of the *D*s of RLs with an increase in the concentration of RLs in solution, and the equality of the *D* values of phenol and RLs at complete solubilization phenol molecules by surfactant micelles.

We used NMR diffusometry to study the process of solubilization of phenol molecules by RL micelles. This method allows one to selectively measure diffusivities of all components in multicomponent liquid mixtures. In this work, it is used to measure diffusion coefficients of phenol, micelles and monomers of surfactants, and water. Selective measurements of *D*s of aqueous solutions of phenol in D_2_O in the presence of RLs were performed, and diffusion characteristics were studied.

The resulting data were analyzed within the framework of a model of two states of phenol in solution, whereby some of the phenol molecules are in the free state in water, and some are in the bound state in surfactant micelles. Next, the solubilization efficiency was calculated, including parameters such as the distribution coefficient of phenol between the micellar (solubilized) and free (in the aqueous phase) states, the molar solubilization ratio (MSR), and the solubilization capacity of rhamnolipid micelles.

In order to refine the concentration dependence, measurements of the phenol *D*s in aqueous D_2_O (without RLs) solutions were made at the phenol concentration from C = 50 g/L, which is close to the limiting solubility of phenol in water, 65 g/L, to *C* = 0.156 g/L, which is close to the condition of infinite dilution. With an increase in the concentration of phenol, diffusivities of both phenol molecules and water molecules decrease. The concentration dependence of the *D*s of phenol molecules at 298 K is described by an equation of the form
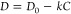
2where *D*_0_ = 0.903 × 10^–9^ m^2^/s is the value of the phenol *D* extrapolated to its infinitesimal concentration in solution and *C* is the concentration of phenol in the solution (g/L), *k* = 2.4 × 10^–12^ m^2^/s·(g/L)^−1^. This value is close to the diffusion coefficient values of phenol in water obtained theoretically by molecular dynamics methods^36,37^ and measured experimentally using the Taylor dispersion technique.^38,39^

Diffusion coefficients of phenol in the presence of RLs were measured at the concentrations of phenol *C*_ph_ = 10, 5, 1, and 0.1 g/L, while concentrations of RLs varied from *C* = 200 g/L to *C* = 0.05 g/L. Initial solutions with maximal concentrations of RLs were carefully mixed and left to equilibrate for at least 2 days. Other solutions were prepared by subsequential dilution. All measurements were made at 298 K.

Diffusion decays of signals of phenol and water in the presence of RLs remain single-exponential, similar to those for individual solutions of phenol.

At the same time, the form of DDs for RLs changes as the concentration of the RLs in the solution changes. In the range of high concentrations *C* = 200 g/L, DDs are single-exponential. This form is maintained as the concentration of RLs decreases down to *C* ≈ 5 g/L. A further decrease in the concentration leads to gradual transition in the form of DD to a nonexponential form. At concentrations of RLs *C* ≈ 0.1 and 0.5 g/L (*C* < CMC), DDs of RLs have distinct nonexponential forms. Such transition from single-exponential forms at high concentrations of RLs to the nonexponential forms at decreased concentrations of RLs was also observed for individual solutions of RLs.

In the author’s opinion, the reason for this phenomenon is the complex composition of RLs, which is a mixture of mono-RLs and di-RLs. RLs are heterogeneous in length and in the degree of branching of hydrocarbon chains, which depends on environmental conditions. It has been noted^40^ that rhamnolipids are a mixture of the homologue species due to variations in the rhamnose units and β-hydroxy fatty acid moieties, mainly including Rha-C10-C10, Rha-Rha-C10-C10, and Rha-C10. The presence in the sample molecules of RLs of different forms and sizes leads to variation in their diffusion coefficients and consequently to the nonexponential DDs at *C*_RLs_ < CMC. At high concentrations of *C*_RLs_ ≫ CMC, micelles predominate in the solution, while the concentration of monomers remains at the level of the CMC.^41^ The compositions of micelles, as well as their sizes, are statistically averaged over all variants of mono- and di-RL molecules. Such a micellar solution consists of micelles of mixed composition, but, on average, the micelles are of the same size. As a result, the diffusion decay of the spin echo signal becomes single-exponential that makes it possible to find the diffusivity of micelles using formula 12. Processing of non-mono-exponential diffusive decays of RLs was performed on the initial sections of the diffusion decays. The slopes of the initial segments of diffusion declines can be used to determine the effective diffusivity of rhamnolipids, assuming that the initial segments of the declines contain information about all fractions and forms of RLs.^42^

**Figure 5:**
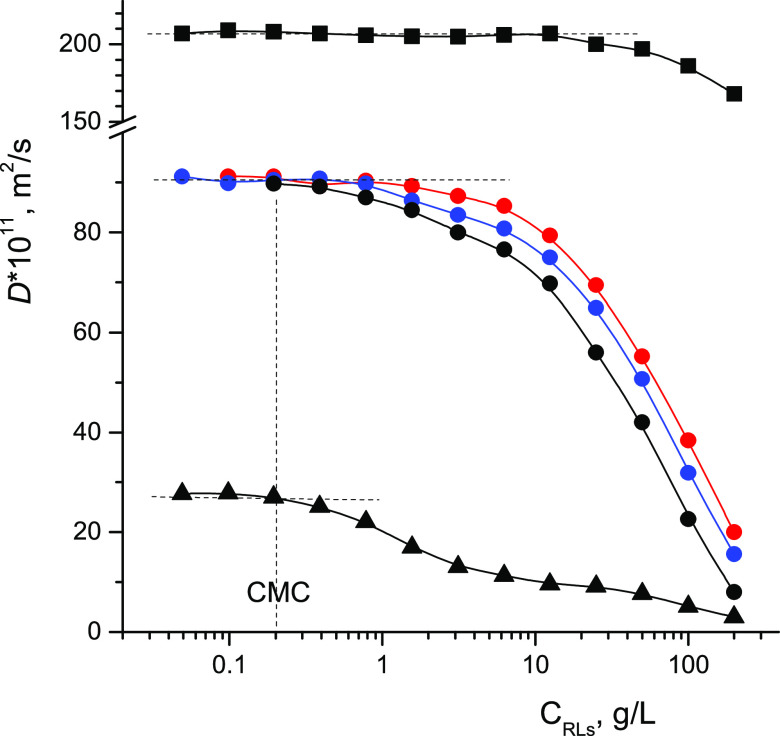
Diffusivities of rhamnolipid (triangle), phenol (circle), and water (square) in aqueous D_2_O solutions in the dependence of RL concentration at concentrations of phenol: 10 g/L (red), 1 g/L (blue), and 0.1 g/L (black). *T* = 298 K.

Figure 5 shows the results of measurements of the *D*s of RLs, phenol, and water molecules depending on the concentration of RLs in solution. *D*s of RLs and water molecules, within the measurement error, do not depend on the presence of phenol in solution. The graph shows the average values from five series of measurements performed at phenol concentrations *C* = 0, 10, 5, 1, and 0.1 g/L. It can also be noted that the *D*s of water molecules do not depend on the concentration of RLs up to *C*_RLs_ ≈ 20 g/L. With a further increase in *C*_RLs_, a decrease in the *D*s of water molecules occurs, which is obviously associated with an increase in the viscosity of the solution at high concentrations of RLs.

*D* values of phenol molecules at *C*_RLs_ < CMC remain constant, do not depend on the concentration of RLs, and slightly decrease in accordance with eq 3, with increasing phenol concentration. At *C*_RLs_ > CMC, the *D* values of phenol molecules sharply decrease (by 1 order of magnitude) with increasing RL concentration, and at high concentrations *C*_RLs_ ≈ 200 g/L, they approach the *D* values of RL molecules.

### 2.3 Efficiency of Solubilization of Phenol by RL Micelles

The efficiency of solubilization, the binding of phenol by micelles, can be determined from the results of measuring the diffusivities of all components in an aqueous solution of surfactant + solubilizate.

Within the framework of the two-state model, solubilizate (phenol) molecules can be inbound (as part of micelles) and free (in water) states. The experimentally measured diffusion coefficient of phenol molecules *D*_ph_ under the condition of fast NMR exchange is a weighted average and can be represented in the form^28^
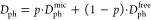
3

where p is the fraction of phenol molecules located in micelles and *D*_ph_^mic^ and *D*_ph_^free^ are *D*s of phenol molecules present in micelles and in the free state in water.

Hence
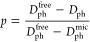
4

Let us assume that the diffusion coefficient *D*_ph_^free^ is equal to the *D* of phenol in solution, measured either in the absence of RLs or at *C*_RLs_ < CMC. *D*_ph_^mic^ can be set equal to the measured *D* of rhamnolipid since the phenol and RL micelles form a single kinetic unit with a common *D* value. At *C*_RLs_ > CMC, the micellar form of the rhamnolipids predominates in the solution, the concentration of the monomeric form remains equal to the CMC,^41^ and the contribution of the monomeric form of the surfactant to the measured *D*s of rhamnolipids is negligible.^43^

**Figure 6:**
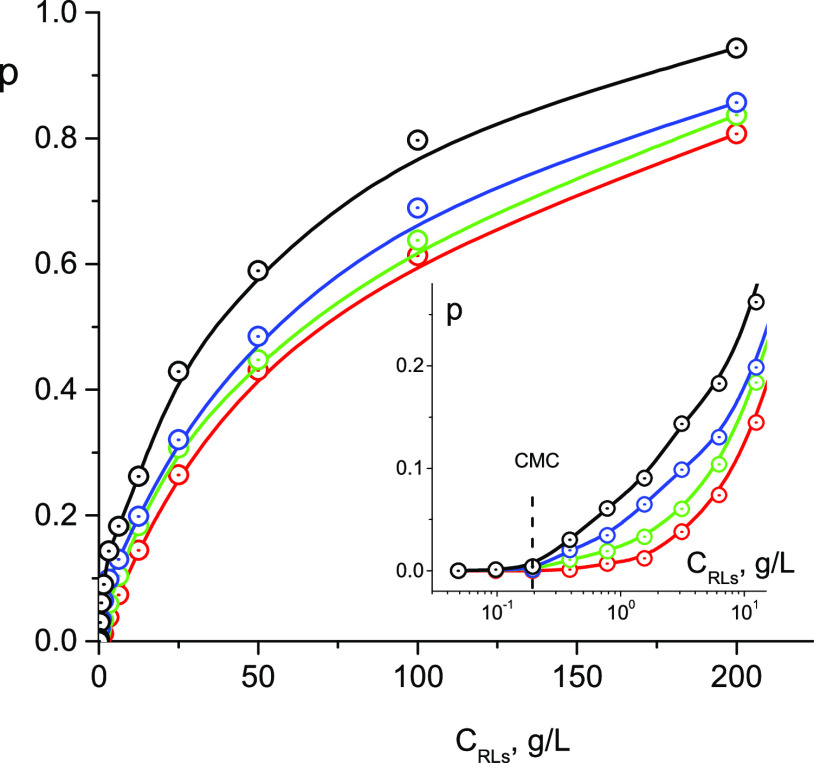
Fraction of solubilized molecules of phenol depending on concentration *C*_RLs_ at concentrations of phenol: 10 g/L (red), 5 g/L (green), 1 g/L (blue), and 0.1 g/L (black). The insert shows a range of low concentrations of RLs. *T* = 298 K.

The results of calculating the proportion of solubilized phenol depending on the concentration of RLs in solution at various concentrations of phenol are shown in Figure 6. The efficiency of phenol solubilization, determined by the relative proportion of phenol molecules bound by micelles, increases with increasing RL concentration and approaches 100% at *C*_RLs_ ≥ 200 g/L. At C_RLs_ concentrations lower or slightly higher than CMC, i.e., in the absence of micelles, there is no solubilization; on the inset to Figure 6 is the region *C*_RLs_ < 0.8 g/L. Solubilization is provided by micelles; with an increase in the concentration of micelles and their sizes, the efficiency of solubilization improves. With an increase in the concentration of phenol in the solution from 0.1 to 10 g/L, the proportion of solubilized phenol molecules decreases, which may be due to the saturation of the solubilizing capacity of micelles at high concentrations of phenol.

Calculations useful for understanding the solubilization efficiency of parameters such as the micelle–water partition coefficient *K*_m_ and molar solubilization ratio MSR were performed. The micelle–water partition coefficient *K*_m_ is equal to the ratio of the number of moles of solubilizate in micelles to the number of moles of solubilizate in the aqueous phase. Or, in other words, the *K*_m_ is equal to the ratio of the fraction of solubilized phenol molecules to the fraction of phenol molecules in the aqueous phase.
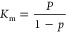
5

The molar solubilization ratio, MSR, is equal to the ratio of the molar concentration of solubilized phenol molecules to the molar concentration of RLs in the micellar state
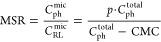
6where *C*_ph_^total^and *C*_RL_^total^ are total molar concentrations of phenol and RLs in solution, respectively.

The solubilization characteristics of RLs with respect to phenol are presented in Table 1.

**Table 1:** Solubilization Characteristics of RLs with Respect to Phenol

	*C*_ph_ = 10 g/L			*C*_ph_ = 5 g/L			*C*_ph_ = 1 g/L			*C*_ph_ = 0.1 g/L		
*C*_RLs_, g/L	*p*	*K*_m_	MSR	*p*	*K*_m_	MSR	*p*	*K*_m_	MSR	*p*	*K*_m_	MSR
0.049	0	0	0	0	0	0	0	0	0	0	0	0
0.098	0	0	0	0.001	0.00	0	0.001	0.00	0	0.001	0.00	0
0.195	0	0	0	0.001	0.00	0	0.001	0.00	0	0.004	0.00	0
0.39	0.001	0.00	0.36	0.011	0.01	2.00	0.02	0.02	0.73	0.030	0.03	0.109
0.78	0.007	0.01	0.83	0.019	0.02	1.13	0.034	0.04	0.41	0.061	0.06	0.073
1.56	0.012	0.01	0.61	0.035	0.04	0.89	0.065	0.07	0.33	0.090	0.10	0.046
3.12	0.038	0.04	0.90	0.060	0.06	0.71	0.099	0.11	0.23	0.143	0.17	0.034
6.25	0.074	0.08	0.85	0.104	0.12	0.59	0.130	0.15	0.15	0.183	0.22	0.021
12.5	0.145	0.17	0.81	0.184	0.23	0.52	0.199	0.25	0.11	0.262	0.36	0.015
25	0.265	0.36	0.74	0.307	0.44	0.43	0.321	0.47	0.09	0.429	0.75	0.012
50	0.431	0.76	0.60	0.448	0.81	0.31	0.485	0.94	0.07	0.589	1.43	0.008
100	0.614	1.59	0.43	0.638	1.76	0.22	0.689	2.22	0.05	0.797	3.93	0.006
200	0.807	4.18	0.28	0.837	5.12	0.14	0.857	6.00	0.03	0.943	16.64	0.003

The solubilization capacity of surfactant micelles is defined as the average number of solubilizate molecules in one micelle or as the number of moles of solubilized molecules to the number of moles of micelles
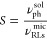
7

The number of moles of solubilized phenol molecules per unit volume (1 L) is equal to
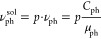
8where *p* is the fraction of the solubilized phenol molecules (eq 5), ν_ph_ is the number of phenol moles in 1 L, and *C*_ph_ and μ_ph_ are the concentration (g/L) and molar mass of phenol, respectively.

The number of moles of rhamnolipid micelles per unit volume is
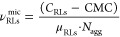
9where *C*_RLs_ is the concentration of RLs in solution, μ_RLs_ is the molar mass of the RLs, and *N*_agg_ is the aggregation number, and the average number of rhamnolipid molecules in one micelle. The molar mass of rhamnolipids depends on the length of their hydrocarbon chains and the number of rhamnose groups in the molecule and varies from 334 to 763 g/mol.^44^ In this work, an unfractionated mixture of mono- and di-rhamnolipids was used, and the molar mass value for it was taken as equal to 650 g/mol.

The aggregation number depending on the concentration of rhamnolipids in solution can be estimated by comparing the volumes of micelles *V* and individual molecules’ volumes. Assuming a spherical shape of micelles and RL molecules, taking the packing factor of molecules in micelles to be 0.74 (dense packing of hard spheres), we obtain
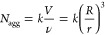
10

Let us rewrite expression 11 taking into account the Stokes–Einstein relation (eq 2) in the form
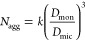
11where *r* and *R* and *D*_mon_ and *D*_mic_ are, respectively, radii and coefficients of diffusion of monomers at *C* < CMC and of micelles of RLs at *C* > CMC.

**Figure 7:**
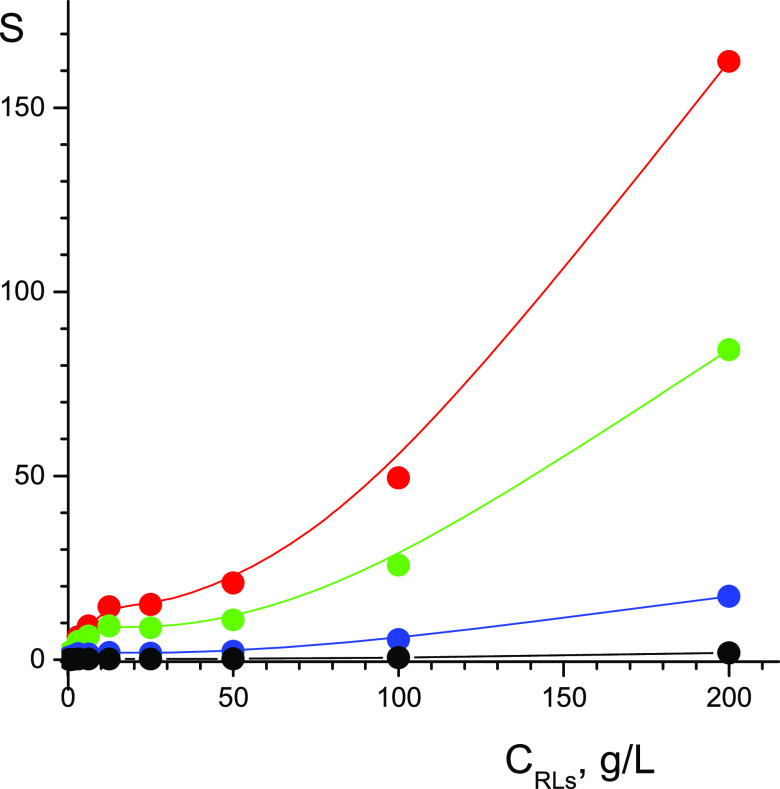
Solubilization capacity of the RL micelles at concentrations of phenol: 10 g/L (red), 5 g/L (green), 1 g/L (blue), and 0.1 g/L (black). *T* = 298 K.

The solubilization capacity of micelles (Figure 7) increases with an increase in the concentration of RLs and the concentration of phenol in solution.

## 3 Conclusions

NMR diffusometry was used to study the solubilization properties of micellar solutions of RLs with phenol as a solubilizate. Concentrations of measurement were *C*_ph_ = 10, 5, 1, and 0.1 g/L, *C*_RLs_ was varied in the range of 200–0.05 g/L, and the temperature was 298 K. Based on the results of selective measurements of diffusivities of the molecules of all components of the solution, phenol, RLs, and water, the CMC values, effective hydrodynamic micelle radii, and solubilization characteristics of RL solutions were determined. Solubilization of phenol by RL micelles manifests itself in the convergence of the *D*s of phenol and molecules of RLs with increasing surfactant concentration and in almost complete equality of the *D*s of phenol and RLs at high concentrations of RLs.

The relative proportion of solubilized phenol molecules bound by micelles increases with increasing RL concentration and approaches 100% at *C*_RLs_ ≥200 g/L. At concentrations of *C*_RLs_ less or slightly higher than the CMC, that is, if there are no micelles in the solution, then there is no solubilization, while the *D*s of phenol molecules remain at the level of the values of the *D*s of phenol molecules in individual aqueous solutions. Solubilization of phenol by RL micelles takes place at all studied concentrations, but it can be noted that with an increase in the concentration of phenol, the proportion of solubilized phenol molecules decreases by approximately 20–30%.

A high solubilizing ability of RLs has been established both with respect to substances of the BTEX group^14^ with low solubility and with respect to phenol, which is moderately soluble in water. Unlike most synthetic surfactants, rhamnolipids have low toxicity and high degree of biodegradability. They are finding increasing use in soil remediation and wastewater treatment.^45^ The results obtained can be used to develop “green” methods for purifying industrial wastewater from organic pollutants and metal ions^46^ using the methods of ultrafine filtration,^47^ extracting and regenerating solubilizate from filters by evaporation, and regenerating RLs for repeated cycles of micellar solubilization.

## 4 Experimental Section

### 4.1 Sample Preparation

Rhamnolipids were purchased from Merck (Germany). It is a mixture of mono- and di-rhamnolipids produced by AGAE Technologies LLC, Corvallis, OR 97333, USA. The content of rhamnolipids in the powder was more than 90%. We did not perform additional purification and fractionation. Chemically pure phenol was used.

All solutions for NMR measurements were prepared in D_2_O (Sigma, degree of isotopic substitution 99.9) at neutral pH (or pD) = 7.44. The use of deuterated water made it possible to exclude the intense line of water from the ^1^H NMR spectra. Solutions for tensiometry were prepared using ordinary distilled water, H_2_O. Before the measurements were taken, the solutions were thoroughly mixed and kept at 298 K for at least 2 days.

### 4.2 Determination of the Ratio of Mono- and Di-RLs

The sample of the study is an unfractionated mixture of mono-RLs, di-RLS, and related products in the form of rhamnose residues and lipids. Analysis and identification of the structure and composition of RLs have been performed by chromatography methods, mass spectrometry, FT-IR spectroscopy, and NMR.^48−52^

**Figure 8:**
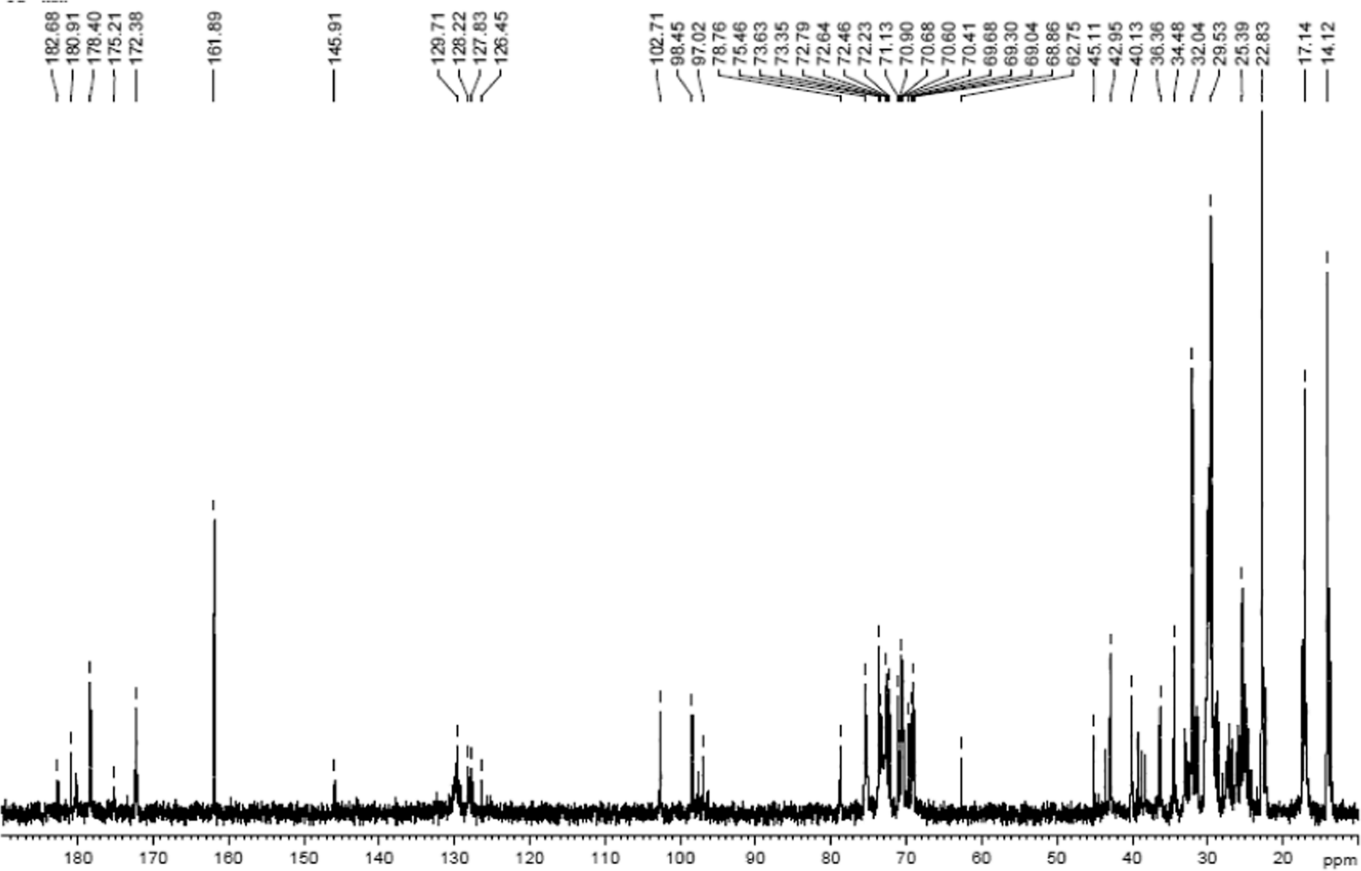
^13^C NMR (100.64 MHz) spectrum of RLs at *C* = 20 g/L, *T* = 298 K, NS = 12 995, and repetition time 5 s.

The powerful tool in multicomponent mixture studies is the ^1^H and ^13^C NMR spectroscopy. To determine the fractional composition in a mixture of mono- and di-RLs, it is necessary to compare the NMR signals of rhamnose and lipid groups in the ^1^H or ^13^C NMR spectra. The use of ^1^H spectra for this purpose is difficult since the most informative line of methyl CH_3_ protons of rhamnose overlaps with the line of methylene CH_2_ protons of lipids.^50,52^ However, in the ^13^C NMR spectrum, the lines of methyl carbons are observed separately,^50^ for both lipids (δ = 14 ppm) and rhamnose groups (δ = 17 ppm) (Figure 8).

Comparison of the intensities of the lines of lipid methyl carbons and rhamnose groups in the ^13^C NMR spectra shows that the studied unfractionated rhamnolipid contains 44% mono- and 56% di-RLs.

### 4.3 NMR Spectroscopy and Diffusometry

^1^H NMR spectra, spin–lattice relaxation times *T*_1_, and self-diffusion coefficients *Ds* of molecules were measured on a Bruker-Avance NMR spectrometer with a resonance frequency of 400 MHz for protons. The self-diffusion coefficients of phenol, water, and rhamnolipid molecules were measured by the pulsed magnetic field gradient method using a stimulated spin echo pulse sequence.^53^ The amplitude of the stimulated spin echo signal is given by
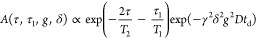
12where *T*_1_ and *T*_2_ are spin–lattice and spin–spin NMR relaxation times, respectively, τ and τ_1_ are time intervals, γ is the gyromagnetic ratio for protons, *g* and δ are amplitude and duration of the pulse field gradient pulses, respectively, *D* is the diffusion coefficient, and *t*_d_ = (Δ – δ/3) is the diffusion time, Δ = (τ + τ_1_).

In the measurements, the magnitude of the impulse gradient was varied up to the maximal value *g*_max_ = 2–15 T/m, while other parameters were not changed: *t*_d_ = 50 ms and δ = 1 ms. The number of scans was set in accordance with the amplitude of the spin echo signal of phenol and rhamnolipid—at high concentrations, NS = 4 was enough, with small NS = 256 and dummy scan DS = 2. The preliminarily measured spin–lattice relaxation time of oxyethylene protons of rhamnolipid was ≈0.5 s and aromatic protons of phenol was ≈6 s. In accordance with this, the time between successive scans was set to RT = 5 s to ensure both the necessary recovery of the NMR signal between successive scans and a sufficiently large number of scans. We processed diffusion decays and determined diffusion coefficients *D*s using Bruker TopSpin 3.5 software.^54^

### 4.4 Tensiometry

The surface tension coefficients were measured on a K9 digital tensiometer (Kruss, Germany) using the ring tear-off method.

## References

[ref1] Jahan R.; Bodratti A. M.; Tsianou M.; Alexandridis P. Biosurfactants, natural alternatives to synthetic surfactants: Physicochemical properties and applications. Adv. Colloid Interface Sci. 2020, 275, 102061 10.1016/j.cis.2019.102061. 31767119

[ref2] Santos D. K. F.; Rufino R. D.; Luna J. M.; Santos V. A.; Sarubbo L. A. Biosurfactants: multifunctional biomolecules of the 21st century. Int. J. Mol. Sci. 2016, 17, 401 10.3390/ijms17030401. 26999123 PMC4813256

[ref3] Kashif A.; Rehman R.; Fuwad A.; Shahid M. K.; Muhammad K.; Dayarathne H. N. P.; Jamal A.; Muhammad N.; Mainali B.; Choi Y. Current advances in the classification, production, properties and applications of microbial biosurfactants–A critical review. Adv. Colloid Interface Sci. 2022, 306, 102718 10.1016/j.cis.2022.102718. 35714572

[ref4] Soberón-Chávez G.; Lépine F.; Déziel E. Production of rhamnolipids by Pseudomonas aeruginosa. Appl. Microbiol. Biotechnol. 2005, 68, 718–725. 10.1007/s00253-005-0150-3. 16160828

[ref5] Müller M. M.; Kügler J. H.; Henkel M.; Gerlitzki M.; Hörmann B.; Pöhnlein M.; Syldatk C.; Hausmann R. Rhamnolipids —next generation surfactants?. J. Biotechnol. 2012, 162, 366–380. 10.1016/j.jbiotec.2012.05.022. 22728388

[ref6] Mańko D.; Zdziennicka A.; Jańczuk B. Thermodynamic properties of rhamnolipid micellization and adsorption. Colloids Surf., B 2014, 119, 22–29. 10.1016/j.colsurfb.2014.04.020. 24840749

[ref7] Li S.; Pi Y.; Bao M.; Zhang C.; Zhao D.; Li Y.; Sun P.; Lu J. Effect of rhamnolipid biosurfactant on solubilization of polycyclic aromatic hydrocarbons. Mar. Pollut. Bull. 2015, 101, 219–225. 10.1016/j.marpolbul.2015.09.059. 26494247

[ref8] Munoz-Cupa C.; Bassi A.; Liu L. Investigation of micellar-enhanced ultrafiltration (MEUF) using rhamnolipid for heavy metal removal from desalter effluent. Can. J. Chem. Eng. 2022, 100, 2322–2330. 10.1002/cjce.24422.

[ref9] Verma S. P.; Sarkar B. Rhamnolipid based micellar-enhanced ultrafiltration for simultaneous removal of Cd (II) and phenolic compound from wastewater. Chem. Eng. J. 2017, 319, 131–142. 10.1016/j.cej.2017.03.009.

[ref10] Abbasi-Garravand E.; Mulligan C. N. Using micellar enhanced ultrafiltration and reduction techniques for removal of Cr (VI) and Cr (III) from water. Sep. Purif. Technol. 2014, 132, 505–512. 10.1016/j.seppur.2014.06.010.

[ref11] Chebbi A.; Franzetti A.; Formicola F.; Ambaye T. G.; Gomez F. H.; Murena B.; De Marco E.; Beltrani T.; Sbaffoni S.; Vaccari M. Insights into rhamnolipid-based soil remediation technologies by safe microorganisms: A critical review. J. Cleaner Prod. 2022, 367, 133088 10.1016/j.jclepro.2022.133088.

[ref12] Liu G.; Zhong H.; Yang X.; Liu Y.; Shao B.; Liu Z. Advances in applications of rhamnolipids biosurfactant in environmental remediation: a review. Biotechnol. Bioeng. 2018, 115, 796–814. 10.1002/bit.26517. 29240227

[ref13] El Zeftawy M. M.; Mulligan C. N. Use of rhamnolipid to remove heavy metals from wastewater by micellar-enhanced ultrafiltration (MEUF). Sep. Purif. Technol. 2011, 77, 120–127. 10.1016/j.seppur.2010.11.030.

[ref14] Arkhipov V. P.; Arkhipov R. V.; Petrova E. V.; Filippov A. Micellar and solubilizing properties of rhamnolipids. Magn. Reson. Chem. 2023, 61, 345–355. 10.1002/mrc.5337. 36840535

[ref15] Babich H.; Davis D. L. Phenol: A review of environmental and health risks. Regul. Toxicol. Pharmacol. 1981, 1, 90–109. 10.1016/0273-2300(81)90071-4. 6764550

[ref16] Guidelines for Drinking-Water Quality: Fourth Edition Incorporating First Addendum; World Health Organization, 2017. 28759192

[ref17] Mohamad Said K. A.; Ismail A. F.; Karim Z. A.; Abdullah M. S.; Hafeez A. A review of technologies for the phenolic compounds recovery and phenol removal from wastewater. Process Saf. Environ. Prot. 2021, 151, 257–289. 10.1016/j.psep.2021.05.015.

[ref18] Quina F. H.; Hinze W. L. Surfactant-mediated cloud point extractions: an environmentally benign alternative separation approach. Ind. Eng. Chem. Res. 1999, 38, 4150–4168. 10.1021/ie980389n.

[ref19] Solubilization in Surfactant Aggregates; Christian S. D.; Scamehorn J. F., Eds.; CRC Press, 2020.

[ref20] Söderman O.; Stilbs P.; Price W. S. NMR studies of surfactants. Concepts Magn. Reson., Part A 2024, 23, 121–135. 10.1002/cmr.a.20022.

[ref21] Edward J. T. Molecular volumes and the Stokes-Einstein equation. J. Chem. Educ. 1970, 47, 261 10.1021/ed047p261.

[ref22] Stilbs P. Automated CORE, RECORD, and GRECORD processing of multi-component PGSE NMR diffusometry data. Eur. Biophys. J. 2013, 42, 25–32. 10.1007/s00249-012-0794-8. 22350080

[ref23] Sánchez M.; Aranda F. J.; Espuny M. J.; Marqués A.; Teruel J. A.; Manresa Á.; Ortiz A. Aggregation behaviour of a dirhamnolipid biosurfactant secreted by Pseudomonas aeruginosa in aqueous media. J. Colloid Interface Sci. 2007, 307, 246–253. 10.1016/j.jcis.2006.11.041. 17182054

[ref24] Wu L. M.; Lai L.; Lu Q.; Mei P.; Wang Y. Q.; Cheng L.; Liu Y. Comparative studies on the surface/interface properties and aggregation behavior of mono-rhamnolipid and di-rhamnolipid. Colloids Surf., B 2019, 181, 593–601. 10.1016/j.colsurfb.2019.06.012. 31202130

[ref25] Kopalle P.; Pothana S. A.; Maddila S. Structural and physicochemical characterization of a rhamnolipid biosurfactant. Chem. Data Collect. 2022, 41, 100905 10.1016/j.cdc.2022.100905.

[ref26] Price W. S. Applications of pulsed gradient spin-echo NMR diffusion measurements to solution dynamics and organization. Diffusion Fundam. 2005, 2, 1–19.

[ref27] Söderman O.; Stilbs P. NMR studies of complex surfactant systems. Prog. Nucl. Magn. Reson. Spectrosc. 1994, 26, 445–482. 10.1016/0079-6565(94)80013-8.

[ref28] Stilbs P. A comparative study of micellar solubilization for combinations of surfactants and solubilizates using the fourier transform pulsed-gradient spin—echo NMR multicomponent self—diffusion technique. J. Colloid Interface Sci. 1983, 94, 463–469. 10.1016/0021-9797(83)90286-2.

[ref29] Lekkerkerker H. N. W.; Dhont J. K. On the calculation of the self-diffusion coefficient of interacting Brownian particles. J. Chem. Phys. 1984, 80, 5790–5792. 10.1063/1.446602.

[ref30] Hardy R. C.; Cottington R. L. Viscosity of deuterium oxide and water in the range 5 to 125 C. J. Res. Natl. Bur. Stand. 1949, 42, 573 10.6028/jres.042.049.

[ref31] Guo Y. P.; Hu Y. Y.; Gu R. R.; Lin H. Characterization and micellization of rhamnolipidic fractions and crude extracts produced by Pseudomonas aeruginosa mutant MIG-N146. J. Colloid Interface Sci. 2009, 331, 356–363. 10.1016/j.jcis.2008.11.039. 19100991

[ref32] Guo Y.; Mulligan C. N.; Nieh M. P. An unusual morphological transformation of rhamnolipid aggregates induced by concentration and addition of styrene: A small angle neutron scattering (SANS) study. Colloids Surf., A 2011, 373, 42–50. 10.1016/j.colsurfa.2010.10.007.

[ref33] Eismin R. J.; Munusamy E.; Kegel L. L.; Hogan D. E.; Maier R. M.; Schwartz S. D.; Pemberton J. E. Evolution of aggregate structure in solutions of anionic monorhamnolipids: Experimental and computational results. Langmuir 2017, 33, 7412–7424. 10.1021/acs.langmuir.7b00078. 28737038 PMC5767468

[ref34] Chen M. L.; Penfold J.; Thomas R. K.; Smyth T. J. P.; Perfumo A.; Marchant R.; Grillo I.; et al. Solution self-assembly and adsorption at the air- water interface of the monorhamnose and dirhamnose rhamnolipids and their mixtures. Langmuir 2010, 26, 18281–18292. 10.1021/la1031812. 21028852

[ref35] Scholz N.; Behnke T.; Resch-Genger U. Determination of the critical micelle concentration of neutral and ionic surfactants with fluorometry, conductometry, and surface tension—a method comparison. J. Fluoresc. 2018, 28, 465–476. 10.1007/s10895-018-2209-4. 29332160

[ref36] Zhang Q.; Han Y.; Wu L.; Zhang C.; Zhu L.; Zhao R. Molecular dynamics simulation of phenol aqueous solution under the impact of an external electrostatic field. J. Chem. Eng. Data 2019, 64, 2259–2265. 10.1021/acs.jced.8b00867.

[ref37] Plugatyr A.; Nahtigal I.; Svishchev I. M. Spatial hydration structures and dynamics of phenol in sub-and supercritical water. J. Chem. Phys. 2006, 124, 024507 10.1063/1.2145751. 16422611

[ref38] Niesner R.; Heintz A. Diffusion coefficients of aromatics in aqueous solution. J. Chem. Eng. Data 2000, 45, 1121–1124. 10.1021/je0000569.

[ref39] Yang X. N.; Matthews M. A. Diffusion coefficients of three organic solutes in aqueous sodium dodecyl sulfate solutions. J. Colloid Interface Sci. 2000, 229, 53–61. 10.1006/jcis.2000.7020. 10942541

[ref40] Li Z.; Zhang Y.; Lin J.; Wang W.; Li S. High-yield di-rhamnolipid production by Pseudomonas aeruginosa YM4 and its potential application in MEOR. Molecules 2019, 24, 1433 10.3390/molecules24071433. 30979013 PMC6480329

[ref41] Lindman B.; Wennerström H. Amphiphile aggregation in aqueous solution. Top. Curr. Chem. 1980, 87, 1–87. 6987777

[ref42] Maklakov A. I.; Skirda V. D.; Fatkullin N. F. Self-Diffusion in Polymer Solutions and Melts; Kazan University Press: Kazan, 1987.

[ref43] Rusanov A. I.; Movchan T. G.; Plotnikova E. V. On the calculation of diffusion coefficients and aggregation numbers of nonionic surfactants in micellar solutions. Colloid J. 2017, 79, 661–667. 10.1134/S1061933X17050118.

[ref44] Müller M. M. Optimization and Characterization of Microbial Rhamnolipid Production from Renewable Resources, Doctoral Dissertation; Karlsruher Institut für Technologie (KIT): Karlsruhe, 2011.

[ref45] Kłosowska-Chomiczewska I. E.; Kotewicz-Siudowska A.; Artichowicz W.; Macierzanka A.; Głowacz-Różyńska A.; Szumała P.; Jungnickel C.; et al. Towards rational biosurfactant design—Predicting solubilization in rhamnolipid solutions. Molecules 2021, 26, 534 10.3390/molecules26030534. 33498574 PMC7864340

[ref46] Aryanti N.; Nafiunisa A.; Giraldi V. F.; Buchori L. Separation of organic compounds and metal ions by micellar-enhanced ultrafiltration using plant-based natural surfactant (saponin). Case Stud. Chem. Environ. Eng. 2023, 8, 100367 10.1016/j.cscee.2023.100367.

[ref47] Moreno M.; Mazur L. P.; Weschenfelder S. E.; Regis R. J.; de Souza R. A.; Marinho B. A.; de Souza A. A. U.; et al. Water and wastewater treatment by micellar enhanced ultrafiltration–A critical review. J. Water Process Eng. 2022, 46, 102574 10.1016/j.jwpe.2022.102574.

[ref48] Raza Z. A.; Khalid Z. M.; Banat I. M. Characterization of rhamnolipids produced by a Pseudomonas aeruginosa mutant strain grown on waste oils. J. Environ. Sci. Health, Part A 2009, 44, 1367–1373. 10.1080/10934520903217138. 20183494

[ref49] Heyd M.; Kohnert A.; Tan T. H.; Nusser M.; Kirschhöfer F.; Brenner-Weiss G.; Franzreb M.; Berensmeier S. Development and trends of biosurfactant analysis and purification using rhamnolipids as an example. Anal. Bioanal. Chem. 2008, 391, 1579–1590. 10.1007/s00216-007-1828-4. 18320178

[ref50] El-Housseiny G. S.; Aboshanab K. M.; Aboulwafa M. M.; Hassouna N. A. Structural and Physicochemical Characterization of Rhamnolipids produced by Pseudomonas aeruginosa P6. AMB Express 2020, 10, 201 10.1186/s13568-020-01141-0. 33146788 PMC7642061

[ref51] Jiang J.; Jin M.; Li X.; Meng Q.; Niu J.; Long X. Recent progress and trends in the analysis and identification of rhamnolipids. Appl. Microbiol. Biotechnol. 2020, 104, 8171–8186. 10.1007/s00253-020-10841-3. 32845366

[ref52] Choe B. Y.; Krishna N. R.; Pritchard D. G. Proton NMR study on rhamnolipids produced by Pseudomonas aeruginosa. Magn. Reson. Chem. 1992, 30, 1025–1026. 10.1002/mrc.1260301019.

[ref53] Stejskal E. O.; Tanner J. E. Spin diffusion measurements: spin echoes in the presence of a time-dependent field gradient. J. Chem. Phys. 1965, 42, 288–292. 10.1063/1.1695690.

[ref54] https://www.bruker.com/en/products-and-solutions/mr/nmrsoftware/topspin.html.

